# Barriers and facilitators for clinical trial participation among diverse Asian patients with breast cancer: a qualitative study

**DOI:** 10.1186/s12905-016-0319-1

**Published:** 2016-07-22

**Authors:** Guek Eng Lee, Mandy Ow, Desiree Lie, Rebecca Dent

**Affiliations:** National Cancer Centre Singapore, 11, Hospital drive, Postal code 169610 Singapore, Singapore; Duke-NUS Graduate Medical School, Singapore, Singapore

## Abstract

**Background:**

Recruitment rates for cancer trials are low for racial/ethnic minorities. Little is known about factors influencing trial recruitment in Asian patients. Our aim is to examine the barriers and facilitators for participation in trials among multi-ethnic Asian women with breast cancer.

**Methods:**

We recruited a convenience sample from consecutive women seen at the National Cancer Centre. Two experienced bilingual (English and Chinese) moderators conducted focus groups to theme saturation. The question guide incorporated open-ended questions soliciting opinions about trial participation and knowledge. Women were first asked if they were willing, unwilling, or still open to participate in future trials. Sessions were audiotaped and transcribed. Transcripts were independently coded for emergent themes.

**Results:**

Sixteen of 103 women approached participated in five focus groups. Chinese, Malay, and Indian participants aged 29 to 69 represented different cancer stages. Five had no prior knowledge of trials. We identified three major themes comprising of 22 minor themes for barriers and facilitators. The major themes were: 1) patient-related, 2) trial-related, and 3) sociocultural factors. Women willing to join trials expressed themes representing facilitators (better test therapy, cost-effective profile, or trust in doctors and local healthcare systems). Women unwilling to participate expressed themes associated with barriers, while women still open to participation expressed themes representing both facilitators and barriers. Malay women were more likely to express themes related to ‘fatalism’ as a barrier.

**Discussion/Conclusion:**

We found that facilitators and barriers to trial participation among Asian women were similar to those previously reported in Western women. Knowledge of trials is limited among women receiving breast cancer treatment. Unique sociocultural factors suggest that approaches customised to local and community beliefs are needed to improve trial participation in minority groups.

## Background

Breast cancer remains the most common cancer affecting women worldwide, accounting for 22.9 % of cancer incidence in women and a mortality of 13.7 % [1]. Advancements in improving patient outcome require rigorous clinical research and application of trial results to clinical care. However, patient recruitment to clinical trials poses a challenge. Recruitment rates to breast cancer trials compared with other cancer trials in particular, are globally low. It has been estimated that only 2–3 % of all breast cancer patients actually participate in clinical trials throughout the course of their treatment [2–4]. Yet, trial participation has the potential to improve clinical outcomes; this is sometimes known as inclusion benefit and has been demonstrated in several studies [5–19]. In addition, treatment modalities and approaches may have different effects on different ethnic and racial groups, making recruitment of diverse populations imperative in trial recruitment [20].

Many factors potentially influence patient recruitment into clinical trials. These can be broadly classified as physician-, patient-, and system-related factors [21]. In a recent study of patients, patient altruism and healthcare professionals’ attitudes were identified as powerful driving forces that motivated patients [22]. Conversely, unease about randomisation, increasing demands of more complex trials, and limited healthcare resources are cited as patient-reported barriers. Other barriers included fear of potential side effects, lack of unawareness of trial opportunities, the idea that clinical trials are not appropriate for serious diseases, and fear that trial participation would have a negative impact on the patient-doctor relationship. Other studies have suggested patient mistrust of the healthcare system and inconvenience of study protocols as additional barriers [23].

These views reflect a Western perspective, with the majority of cited studies being from the United States and Canada. There is a need for understanding such factors in diverse global populations, especially in an Asian setting, since clinical trials now trend toward international multi-site settings with rapid growth in Asian countries. In particular, the recruitment rate of patients into clinical trials in Asia is much lower than in other parts of the world [24]. A clearer understanding of barriers and facilitators in these settings will permit implementation of interventions to improve future trial recruitment.

Singapore is an island nation of five million in Southeast Asia, with a diverse, mainly English-speaking population consisting of ethnic Chinese, Indian, Malay, and a minority of other races [25]. This unique multicultural setting provides a valuable opportunity to elicit views representative of different Asian subgroups. We conducted a focus group study of different ethnic groups to examine barriers and facilitators to cancer trial recruitment in patients, with particular focus on multi-ethnic Asian women with breast cancer.

## Methods

### Study setting

The study was conducted at the National Cancer Centre Singapore (NCCS), a regional centre in Asia for the research and treatment of all cancers. The NCCS treats 75 % of cancer patients in Singapore, while the remaining 25 % are cared for in other private and public hospital settings. Yearly outpatient attendance at the NCCS is more than 130,000, and new cancer cases number more than 6,000 per year [26].

### Patient recruitment

This qualitative study was approved by Centralised Institutional Review Board, Singhealth research. Between March and May 2013, NCCS patients attending the breast clinic of four oncologists were recruited by three medical students. The recruiters approached potential focus group participants in the waiting room from Monday to Friday between 9 am and 5 pm. The sample recruited consisted of female breast cancer patients older than 21 years, capable of both providing informed consent, and attending the focus group independently. Informed consent was obtained in the outpatient setting.

### Focus group moderator guide and procedures

We conducted a literature review to identify information about the factors that influence patient recruitment into clinical trials. Seven studies were identified from the review and the information obtained was used as the basis for the moderator guide [20–23, 26–28]. We used open-ended questions framed in a locally and culturally appropriate context (Table 1) to encourage discussion and exploration of pertinent barriers and facilitators.

**Table 1:** Moderator question key and guide for focus groups on barriers and facilitators to trial participation

1) Prevalent knowledge, understanding, and beliefs about clinical trials	
	• Have you heard about clinical trials?
	• What do you understand about clinical trials?
	• How do you feel about clinical trials?
2) Willingness to participate in clinical trials	
	• Open to listening
	• Cautious
	• Waiting to say ‘No’
3) Specific probes into facilitators and barriers to participating in clinical trials	
	What are the non-trial related factors that you will consider?
	• Your current status of health?
	• Ability and willingness to adhere to trial schedule?
	• Priorities?
	• Opinions of family and friends?
	What are the trial related factors that you will consider?
	• How do you feel about the idea of randomisation?
	• How do you feel about the new treatment?
	• How do you feel about the closer monitoring?
	• How do you feel about more blood tests?

We conducted five focus groups over three months, with each group consisting of three to five participants. Trained bilingual (English and Chinese) primary and a secondary moderator were present for each group. The sessions were audiotaped and transcribed in the original language. Chinese text was then translated into English for coding purposes. Translation was done with the help of medical students during the process of transcription from audio files. Each focus group session comprised three parts. First, the primary moderator explained what a clinical trial was; this determined existing levels and sources of knowledge about, as well as prevalent attitudes toward trials. Following that, we presented participants with a mock informed consent form (ICF) for a hypothetical clinical trial in order to provide uniform basic information about trial recruitment procedures; this simulated the type of information provided during a real-life enrolment. The second part of the focus group session assessed participants’ initial willingness and factors that influence their willingness and decisions to take part in trials. The discussions were initially open-ended. After no new factors were spontaneously suggested by participants, the primary moderator asked participants about factors identified from our literature review. These factors were included as standardised moderator probes in the moderator question guide (Table 1). The final part invited participants to suggest the ideal presentation of trial information to potential participants.

The primary moderator was responsible for leading the session while the secondary moderator took field notes. After every focus group discussion, debriefing between the two moderators took place to review and improve the question guide and the process for subsequent focus groups. The aim was to complete five focus groups or until theme saturation was reached, whichever came first.

### Data analysis and coding

The primary data source was the typed transcripts for each focus group. The text was coded using content analysis procedures [29]. Transcription and coding were completed within three days of each focus group so as to identify issues that could be addressed in subsequent focus groups. If indicated, the moderator guide was modified. In this way, the first focus group discussion also served as a pilot.

Each transcript was independently coded by the two primary coders. A codebook with themes and supporting quotes was then created by the primary coders, who met to discuss themes after each set of transcripts was analysed. If the agreement in themes was less than 90 %, two secondary coders were available to adjudicate. Major and minor themes were derived from a combination of pre-set questions in the moderator guide as well as from unique data in the transcript.

All transcripts were then recoded using the finalised version of the codebook. A summary of all the themes was generated and similar themes were grouped into broader and more abstract categories. We used qualitative data analysis software (NVivo 9, QSR International), to tabulate theme frequency using the codebook as a basis for counting and sorting the minor themes.

## Results

A total of 103 women undergoing breast cancer treatment were approached. Of these, 28 (27 %) verbally agreed to participate in focus groups. Sixteen (16 %) subsequently attended the groups, and 12 were unable to do so due to scheduling conflicts. Five focus groups were conducted between March and May 2013. Details of participants’ characteristics are summarised in Table 2.

**Table 2:** Patients’ demographics for five focus groups

	Numbers (Total *n* = 16)
Age	
29–50	3
51–69	13
>70	0
Race	
Chinese	12
Malay	4
Indian	0
Education level	
GCE ‘O’ level	3
GCE ‘A’ level	2
Polytechnic Diploma	1
University Degree	2
Unknown	8
Stage of breast cancer	
I	4
II	5
III	4
IV	3

Results are presented in three parts. Part 1 reports prevalent knowledge and attitudes of participants toward clinical trials. Part 2 discusses 22 minor themes about barriers and facilitators under three major themes (patient, trial and sociocultural). Part 3 summarises participant suggestions for ideal presentation of trials to potential participants.

Agreement on themes was greater than 90 % between the two primary coders, and no adjudication was needed. A total of three factors with 22 minor themes for barriers and facilitators emerged in the final analysis (Table 3). The major factors are organised and presented as being related to the individual or patient, to the trial, and to broader sociocultural factors.

**Table 3:** Minor theme frequency for three factors, organised as facilitators and barriers to clinical trial participation

Minor themes for three major factors (patient-, trial- and sociocultural-related)	Number of respondents
Patient-related factors	
Facilitators:	
• Opinion of family and friends	12
• If test therapy is last resort (late stage/failure of current therapy)	6
• If overall benefits outweigh risks	2
• If general health is better	2
• Altruism	6
Barriers:	
• Previous bad experiences with doctors, trials, and drugs	3
• Conservative attitude towards risk-taking	2
• Unique health situation	2
• Cancer is a serious illness	1
Trial/protocol-related factors	
Facilitators:	
• Hope that test therapy is better treatment option	9
• Closer monitoring	3
• Lower cost of treatment	3
• Trust that care is adequate during trial procedure	2
Barriers:	
• Risk of test therapy/Uncertainties inherent in investigational drugs	13
• Perceived risk associated with randomisation	5
• Additional visits required	5
• Systemic therapy	2
• Added stress from trials	2
• Additional tests required	1
Broader Socio-cultural/System-related factors	
Facilitators:	
• Trust in physicians	7
• Trust in drug development/regulation and healthcare system	8
Barriers:	
• Fatalism	1

### Part 1: Prevalent knowledge and attitudes toward clinical trials

Five participants reported that they had not previously heard of the term “clinical trials”, while two were previously or currently enrolled in trials. The remaining participants had heard of clinical trials from various sources, including newspapers, health magazines, the Internet, and friends or relatives previously enrolled in trials. However, the level of understanding concerning clinical trials was low. Eighty percent of the participants asked the session moderator for more information about trials. A range of attitudes was expressed about clinical trials from anxiety to disinterest to curiosity. Quotes reflecting these attitudes include:

*“I am scared” or “I am nervous” (about joining a trial), “doesn’t really concern me” or “unless the doctor tells me that I need this, I will not be bothered”.*

Conversely, an optimistic attitude to joining clinical trials was also expressed, as evidenced by the following quote:

*“I find it is okay (to join clinical trials), especially if it can help future generations.”*

### Part 2: Factors affecting decision whether to participate in clinical trials

In aggregate, participants offered many factors affecting their decisions to join clinical trials. We categorised them broadly into three areas: factors related to the individual patient, factors related to clinical trial characteristics, and factors related to broader systemic and cultural issues. The minor themes associated with each area are summarised in Table 3.

#### Factors related to the individual

Minor themes associated with this major theme fell into the categories of facilitators and barriers. For facilitators, an important minor theme was‘the opinion of family and friends’. Twelve participants cited the opinions of their friends and family as an important facilitator (if the opinions were positive) or barrier (if the opinions were negative).

A second minor theme was ‘test therapy is last resort’. In other words, participants were willing to participate when no other options were available, for example, at later/terminal stages of cancer. This theme was expressed by six participants, showing that trial therapy as a last resort is often a facilitator and is regarded as a last-ditch attempt; in the related minor theme of ‘if overall benefits outweigh risks’, two participants stated that they would participate in trials if the perceived overall benefit outweighed risks.

The following quotes exemplify the minor themes identified above:

*“If I am stage four, I do not mind trying.”*

“*If I am in an early stage and curable, why would I try a new drug?”*

*“I think it will come to a certain stage where you have no other option, when you face the wall; then you will go for the opportunity.”*

The minor theme of ‘if general health is better’ emerged as a facilitator, as participants expressed that this allowed better tolerance of trial drug side effects. ‘Altruism’ emerged as a minor theme among participants who saw trials as a way to advance treatment for others, exemplified by these quotes:

*“I would go for it. If nobody takes a step, then how will medicine advance?”*

*“I do not mind joining the trial if I can use the new medicine and help others at the same time.”*

Barriers to trial participation can be classified into four minor themes. ‘Previous bad experiences with doctors, trials and drugs’, was a prominent minor theme. One participant described a negative experience with the primary healthcare system and a public hospital institution; her diagnosis of cancer was delayed, and she felt that this subsequently led to a near-death experience. As such, she did not want to consider any clinical trials. She said:

*“I trusted the doctors and this is the end result (referring to late diagnosis). Now, unless I am very sure, I will not take part in clinical trials.”*

‘Conservative attitude toward risk-taking’ was a third minor theme representing the concept of personality traits being a barrier to trial participation. For example, two participants reported that their conservative attitudes prevented them from participating in a trial, while another said that the opportunity cost of participating in trials was greater for cancer compared with other chronic diseases such as diabetes. These quotes represent some of these views:

*“I am the sort who will go for lower gains but definite returns.”*

On the converse side for the notion of ‘risk-taking’, another participant stated:

*“I want to try something new; I don’t want to stick to something old and not improved.”*

The minor theme of ‘unique health situation’ also emerged, representing the participant’s perception that poor health is a reason to not participate in trials. The following quotes are examples of this minor theme:

*“If my health is not good, I will not join.”*

*“People with different body constitutions will get different effects from the medication.”*

Finally, ‘cancer is a serious illness’ was a minor theme for barriers to trial participation. Patients felt that cancer is a life-threatening disease; hence they will be more cautious when considering trial participation for cancer research. This can be exemplified by the following quotes:

*“If it is a trial for something less life-threatening, maybe I will consider.”*

*“It is not like normal, like high blood pressure medication. High blood pressure is very common and if I try a new medication for that I can still do monitoring myself.”*

#### Factors related to clinical trials

Facilitators for trial-related factors include the hope that test therapy is a better option. Nine participants indicated that the hope of the test therapy improving their condition was a prominent facilitator to participate in trials. The second minor theme is the lower cost of treatment. Three participants quoted lower costs of treatment when they participate in trials as an important facilitator. Other pertinent facilitators which contributed to the third minor theme include closer monitoring during the trial as well as trust that care is adequate during the trial procedure.

Trial conduct is important to participants. Clinical trials conducted in an ethical manner, with full description of compensation details should they suffer injury during the trial process, was the fourth minor theme appealing to trial participation. Two participants cited trust that care is adequate during trial procedure as facilitators. While closer monitoring was cited as a barrier for some participants, three participants felt that this is an important facilitator for trial participation.

A frequent minor theme for barriers was ‘fear of investigational drugs’. This includes both the known and unknown risks inherent in investigational drugs. Thirteen participants cited this as the predominant barrier to joining clinical trials.

*“Chemotherapy already has so many side effects, let alone the new drug.”*

Participants who were unwilling to join trials felt that there was a worse risk-benefit ratio to joining trials as there may be unanticipated side effects combined with a perceived lack of efficacy data on the investigational drug.

*“There is uncertainty with new drugs; there is no guarantee.”*

*“Seems like there is a lot of uncertainty, and the side effects seem pretty bad.”*

Another minor theme highlighted as a barrier includes systemic versus local therapy. Discomfort with investigational drugs was more pronounced if the treatment were systemic compared to local/topical treatment.

Another minor theme cited by five participants is the perceived risk associated with randomisation. Randomisation was likened to “taking a chance”. Participants who were unwilling to join trials were uncomfortable with the idea of randomisation; they felt that not being allowed to choose treatment options was equivalent to leaving their life to chance. One participant confessed that she would consider dropping out from a clinical trial if she found out that she was not taking the treatment medication.

*“I want to decide for myself and not let luck or others decide for me.”*

*“I only want to take part if I can choose.”*

Some protocol-specific factors were important to select participants. Participants who were unwilling to join trials reported that frequent blood tests or clinic visits were deterrents. They felt that additional monitoring added to the stress of therapy

#### Broader socio-cultural/system-related factors

Trust in physicians as well as local governance were the two most common minor themes quoted as facilitators (*n* = 15).

More than half the participants were more willing to join trials if they were introduced by their own physicians. When probed further, most agreed that trust in their primary physician was the main motivator.

*“I trust my doctor, which is why I leave my life in his hands.”*

*“I’ll discuss with my doctor first. And if my doctor recommends it then I will try (laughs).”*

Participants willing to participate in trials expressed trust in the drug development process; trust in government legislation governing trials also played a part in encouraging patients to join clinical trials. Amongst the patients interviewed, all expressed trust in the local healthcare system.

*“If the government approves, and since our medicine is so advanced, then will believe that it is safe. You see even foreigners come here for treatment.”*

A conservative attitude as well as fatalism was the most common minor themes associated with barrier under broader socio-cultural factors. Two participants also cited cultural differences as a reason for their reluctance to join trials. One patient elaborated: *“We are Asian, you see; our mind-set is quite conservative, so we are not as daring.”* Another participant commented that “*because of our upbringing, we are not as adventurous*.” It seems that Asian culture, where patients are less adventurous and more cautious, is one of the main themes that act as a barrier towards trial participation in this part of the world.

### Part 3: Ideal presentation of trial information

In an attempt to understand and improve trial recruitment rates, we encouraged patients to discuss their ideal setting for clinical trial discussions. The suggestions put forth by participants can serve as a platform for the implementation of changes to aid in trial recruitment.

Firstly, trials should be introduced by either their primary physician, or the person who is in charge of the trial and is knowledgeable about it. However, subsequent elaboration could be carried out by anyone who was familiar with the trial, not necessarily doctors.

*“I will think that if the doctor introduces the trial first I would consider (you know what I mean). Your own doctor will know your health condition better than anyone.”*

*“And maybe after the doctor has already explained to me, then the research assistant can give further explanations.”*

Secondly, the setting of trial discussions should be formal, such as in a room or clinic and not along clinic corridors or waiting areas. A minority of patients (25 %) felt that the setting was not a priority, as long as they were approached in a polite manner.

Thirdly, ICFs (Informed Consent Forms) should be simplified by using simpler language and more pictures to aid in delivery of information.

*“I also study TCM (Traditional Chinese Medicine). A lot of the time, the instructions were given in pictorial form so that it helps with understanding.”*

Lastly, visual aids such as video recordings can be played at waiting areas to increase exposure and awareness of clinical trials.

*“Use of videos will help us to understand more.”*

## Discussion

Our study confirms findings from previous studies in identifying themes for both barriers (fear and uncertainty about new drugs, time restrictions, and mistrust of system) and facilitators (late stage disease, altruism, trust in physicians, and prior positive experience with trials) [21, 22]. We found new themes for both barriers (cultural beliefs for example, ‘fatalism’, and negative opinions of friends/relatives); and facilitators (optimism with improved health, and financial incentive, such as not having to pay for new drugs) among our participants. Our stratified thematic analysis found that participants willing to join clinical trials were more likely to cite trust in doctors (43 %) and local governance (50 %), as well as the hope of the test therapy being a better treatment option (56 %) as facilitators. Participants who were less willing to join clinical trials were more likely to cite barriers such as uncertainties inherent in investigational drugs and risks of trial therapy (81 %) as well as inconveniences associated with the trial set-up (31 %), and the perceived risks of randomisation (31 %) (Table 3). Respondents who expressed unwillingness to join clinical trials also emphasised cultural and personal barriers, lack of awareness about the trials, and the absence of facilitators; while those who had no opinion about joining clinical trials cited both barriers and facilitators equally.

Our study is unique in the recruitment of a diverse group of women (for age, ethnicity, language, and breast cancer stage) with clearly expressed opinions about their willingness to join clinical trials. We were able to reach theme saturation for both Chinese and Malay ethnic groups. While the cultural factors the women expressed may be unique to our setting, we posit that similar factors will be found among women from other cultural settings. This is especially so in the Asian context where decision-making often involved community and family as strong social influences. In addition, we were able to stratify the coding to three subgroups because participants were asked about their willingness to participate in trials, enriching the analysis. We believe that the data derived from this analysis is transferable to other settings with similarly diverse patients.

Although the total number of participants [16] is small, they represent the full profile of patients from the NCCS, in which they were not significantly different from non-participants (Table 2). Our data analysis suggests that having more focus groups would not have yielded more themes, as there was theme saturation after analysis of data from five focus group discussions. Since the study involved only women, the findings may not transfer to men for whom other barriers and facilitators may operate. Although the study involved only women with breast cancer, the responses and themes elicited suggest that the opinions were not unique to breast cancer patients.

## Conclusion

Clinical trial recruitment is often limited by challenges of minority recruitment (barriers to recruiting, for example, urban African American women into research studies in community settings), and a clear understanding of the cultural issues and barriers is needed to increase recruitment [22]. Our findings have implications for minority recruitment into cancer trials and suggest appropriate interventions that may improve recruitment. For example, patient autonomy is often the guiding principle in decision-making and trial recruitment often involves consent from the patient only. But in some Asian cultures where decision-making involves the community and family, influencing attitudes and opinions in the larger community may be an important first step before patient recruitment. In our study, both Chinese and Malay women expressed a lack of knowledge about the purpose and processes of clinical trials and inadequacy about making decisions. Patient education may need to be more intense and address literacy as well as cultural barriers to improve recruitment. Trust in their own (but not in other unknown) physicians was a dominant theme among participants, which suggests that recruitment might be more successful if conducted through the patients’ own primary care physicians and oncologists. Our findings suggest that researchers need to pay more attention to both individual and community cultural factors when considering recruitment strategies or training recruiters.

In conclusion, barriers and facilitators to trial participation are similar among multi-ethnic Asian women as those previously reported among Western women. Additional cultural and personal factors among Asian women suggest new potential strategies for enhancing future recruitment. More information is needed on the attitudes of men and of patients of both genders with cancer types other than breast cancer. Future studies will identify effective patient, physician, and community educational interventions to improve clinical trial participation among cancer patients.

## Abbreviations

ICF, informed consent form; NCCS, National Cancer Center Singapore; TCM, Traditional Chinese Medicine
